# Integration of Gestalt Therapy with Evidence-Based Interventions for Borderline Personality Disorder—Theoretical Framework and Clinical Model

**DOI:** 10.3390/brainsci15101109

**Published:** 2025-10-15

**Authors:** Enrico Moretto, Roberta Stanzione, Chiara Scognamiglio, Valeria Cioffi, Lucia Luciana Mosca, Francesco Marino, Ottavio Ragozzino, Enrica Tortora, Raffaele Sperandeo

**Affiliations:** 1 SiPGI–Postgraduate School of Integrated Gestalt Psychotherapy, 80058 Torre Annunziata, Italy; dr.robertastanzione@gmail.com (R.S.); chiarasco78@libero.it (C.S.); dr.valeriacioffi@gmail.com (V.C.); moscalucialuciana@gmail.com (L.L.M.); francescomarino119@hotmail.it (F.M.); dr.ottavioragozzino@gmail.com (O.R.); enricatortora1604@gmail.com (E.T.); 2 Department of Neurosciences Reproductive and Odontostomatologic Sciences, University of Naples “Federico II”, 80131 Naples, Italy; raffaele.sperandeo@gmail.com

**Keywords:** gestalt therapy, borderline personality disorder, DBT, schema therapy, therapeutic integration, phenomenology

## Abstract

Background/Objectives: Gestalt therapy traditionally opposes categorical diagnostic labelling due to its fundamental inconsistency with phenomenological and process-oriented ontology. However, this epistemological rigour can limit integration with structured evidence-based interventions for complex personality organizations such as Borderline Personality Disorder (BPD). Despite the evidence base for DBT and Schema Therapy in treating BPD, these approaches may inadvertently minimize the lived phenomenological experience and organismic wisdom central to recovery. Meanwhile, Gestalt therapy’s anti-diagnostic stance limits its integration with structured evidence-based protocols. This paper proposes a hybrid theoretical model that addresses this gap by integrating the clinical epistemology of Gestalt therapy with Linehan’s biosocial theory of Dialectical Behaviour Therapy (DBT) and schema-focused interventions, while preserving the core principles of Gestalt. Methods: we present a model of theoretical integration that draws on Gestalt contact theory, the four modules of DBT (mindfulness, distress tolerance, emotional regulation, interpersonal effectiveness) and the experiential techniques of Schema Therapy. The integration focuses on the dialectic of acceptance and change, which mirrors Gestalt’s paradoxical theory of change. The proposed framework preserves the non-protocol dimension of Gestalt therapy while incorporating the pragmatic utility of DBT and Schema Therapy. Results: key conceptual contributions we propose include: (1) theorizing the “Draft Self” as the object and subject of therapeutic work, (2) integrating mindfulness and grounding as embodied processes within live Gestalt experiments, (3) activation techniques to explore the identity fragmentation endemic to BPD. Conclusions:his integration offers a coherent, embodied, and process-oriented framework for understanding and treating BPD that validates patients’ lived experience, mobilizes evidence-based interventions, and opens up meaningful intertheoretical dialogue.

## 1. Introduction

Borderline Personality Disorder (BPD) affects approximately 1–2% of the general population and is associated with substantial functional impairment, high rates of self-harm, and significant healthcare utilization [add recent epidemiological reference]. While evidence-based treatments such as Dialectical Behaviour Therapy (DBT) and Schema Therapy have demonstrated efficacy in reducing suicidal behaviour and improving emotion regulation, clinical outcomes remain heterogeneous, and many patients continue to experience identity confusion and existential distress even after symptom reduction.

Current evidence-based approaches, while structured and effective, have been critiqued for potentially overlooking the phenomenological depth and organismic self-regulation capacities emphasized in humanistic-experiential traditions.

The clinical Gestalt approach to borderline personality organization favours phenomenological exploration over diagnostic categorisation [1]. Isadore From’s concept of the “Draft Self”—a fragile and provisional construct that borderline individuals maintain in order to preserve identity coherence amid internal fragmentation—provides a framework for therapeutic engagement [2]. This “draft” represents not a fixed structure of the self but a precarious gesture towards coherence in the face of overwhelming uncertainty.

Recent empirical research has provided a nuanced understanding of the complex etiology of BPD. A large-scale study of 602 participants that examined dissociative experiences and temperamental-characterological traits showed that dissociative symptoms had greater predictive weight (89% significance) for the diagnosis of BPD than characterological traits alone [3]. This finding reinforces clinical observations that many individuals with BPD report histories of trauma, particularly sexual abuse [4].

However, trauma alone is insufficient to explain the development of BPD. Only individuals whose temperamental and characterological traits fall at certain extremes—such as high harm avoidance and low self-direction and cooperativeness—appear particularly vulnerable to translating traumatic stress into dissociative symptomatic patterns and identity disruption [5]. This complex interaction supports a model in which BPD emerges from non-linear and recursive processes involving constitutional vulnerabilities, traumatic environmental experiences, and dissociative coping mechanisms.

Network theories of psychopathology have argued that such disorders are not the product of single causal pathways but dynamic constellations of symptoms that reinforce each other over time [6]. Gestalt therapy responds to this complexity by cultivating awareness of how dissociation [7], identity diffusion and relational turbulence manifest themselves in the immediacy of contact.

This paper aims to: (1) develop a theoretically coherent integrative framework that preserves Gestalt therapy’s phenomenological rigour while incorporating the structured interventions of DBT and Schema Therapy; (2) articulate how the concept of the ‘Draft Self’ can serve as a unifying clinical focus; (3) propose practical session structures and therapeutic domains that operationalize this integration; and (4) identify the epistemic challenges and training requirements inherent in such hybrid practice. We present this as a conceptual model requiring future empirical validation rather than an established treatment protocol.

## 2. Methods: Approach to Theoretical Integration

This paper presents a conceptual synthesis rather than empirical research. The integrative framework was developed through: (1) systematic narrative review of theoretical literature on Gestalt therapy, DBT, and Schema Therapy; (2) identification of conceptual resonances and epistemological tensions between approaches; (3) clinical workshop discussions conducted at the Puglia Gestalt Summer School 2025, where preliminary integration strategies were explored with practicing clinicians; (4) iterative refinement based on case consultation experiences within our training institute.

The session structures, therapeutic domains, and clinical illustrations presented represent theoretical proposals derived from this synthesis process, informed by clinical observation but not systematically tested. These should be understood as hypothetical frameworks requiring empirical validation through case studies, process research, and eventually comparative outcome trials.

## 3. From Diagnostic Label to Process Field

Borderline Personality Disorder is characterized by pervasive emotional instability, identity diffusion, impulsivity and patterns of interpersonal chaos. Kernberg’s influential model conceptualizes these phenomena as arising from three fundamental intrapsychic processes: identity diffusion, reliance on primitive defences (splitting and projective identification) and fluctuations in reality testing [8].

In Gestalt therapy, these patterns are reformulated as dynamic and observable disruptions of contact and self-regulation: projection, confluence, retroflection, and deflection. Diagnosis becomes a process rather than a label, monitoring real-time indicators that include:

• Dysregulated contact boundaries (over-merging or withdrawal);
• Somatic signals of splitting: muscle rigidity, frozen gaze, shallow breathing;
• Behaviours that interfere with therapy: chronic tardiness, sudden anger, seduction or avoidance, seen as emerging field phenomena rather than resistance.

### 3.1. The Draft Self as a Therapeutic Focus

To preserve the draft of the Self, we can feel the therapeutic presence in contact with others with this type of attitude towards the patient.

• Clear boundaries: Therapists maintain their embodied individuality during relational storms (“I am here and I am not consumed by your accusations”).
• Curious engagement: Genuine interest in how the patient enacts the draft fosters trust and co-presence.
• Embodied exploration: Inviting patients to trace bodily sensations as they enact the draft deepens contact with emerging individuality.
• Affirmative moment-to-moment dialogue: This micro-process respects the function of the draft, allowing subtle changes that suggest emerging integration.

The draft of the self thus becomes both the object and subject of therapeutic work: object as what the patient presents; subject as living, emerging and transformable within the field of contact.

### 3.2. Epistemological Reconciliation: Pragmatic Use of Diagnostic Categories

The integration proposed here requires explicit acknowledgment of an epistemological tension: Gestalt therapy’s foundational rejection of categorical diagnosis appears incompatible with the medical-model origins of DBT and Schema Therapy. However, we argue for a pragmatic rather than ideological stance toward diagnostic frameworks.

We adopt the diagnostic category of BPD not as an ontological truth about the patient’s being, but as a provisional organizational map—a shared language that facilitates communication with other clinical systems, insurance providers, and research communities. This pragmatic instrumentalism allows us to interface with evidence-based protocols without reifying the diagnosis as the patient’s essential nature.

In practice, this means: (1) using BPD criteria as initial phenomenological indicators rather than fixed traits; (2) remaining vigilant against the pathologizing effects of diagnostic language in the therapeutic relationship; (3) continuously returning attention to the lived, emergent experience rather than diagnostic confirmation. The diagnosis serves as a starting point for clinical conversation, not its endpoint. This approach resonates with critical psychiatry perspectives that view diagnostic categories as socially constructed tools rather than natural kinds.

## 4. Integration: Gestalt Therapy, DBT and Schema Therapy

### 4.1. Integration of Dialectical Behaviour Therapy (DBT)

Dialectical Behaviour Therapy (DBT), developed by Linehan, is one of the most influential contemporary treatments for BPD, demonstrating significant reductions in suicidal behaviour, hospitalisations and BPD symptoms [9,10]. Its conceptual core lies in biosocial theory, which posits that chronic emotional dysregulation stems from the interaction between biological vulnerabilities (increased limbic reactivity) and invalidating environments that punish, trivialize, or ignore emotional expression.

Linehan has operationalised this model in a structured treatment with four primary modules, each adaptable within a Gestalt framework:

Mindfulness: Learning to observe thoughts and emotions without judgement, bringing attention back to the present moment.

Gestalt adaptation: Include guided body scans of 2–3 min in sessions to anchor attention and raise awareness of physiological arousal.

Tolerance to suffering: Cultivate the ability to survive emotional crises without self-harm or impulsivity.

Gestalt adaptation: Experiment with changes in temperature or position, body activations, changes in breathing, continuum of awareness and progressive relaxation practised in session.

Emotional regulation: Increase understanding and modulation of emotional responses.

Gestalt adaptation: Map somatic triggers and emotional vulnerabilities with phenomenological reformulations; create experiments to observe different emotions using different media (artworks, books, music, etc.).

Interpersonal effectiveness: Develop assertive communication and the ability to set boundaries.

Gestalt adaptation: Use chair work or group work to recognize the integration between polarities: assertive vs. destructive aggression, empty vs. full, fear vs. excitement.

#### 4.1.1. Integration of Schema Therapy

Schema Therapy, developed by Jeffrey Young [11], is a therapeutic approach that combines evidence-based cognitive-behavioural techniques with elements of interpersonal, experiential and psychodynamic therapies, specifically designed for the treatment of personality disorders and other complex issues. The principles of this approach concern the following.

#### 4.1.2. Early Maladaptive Schemas

Early maladaptive schemas are emotional, cognitive, and behavioural patterns that arise at an early age when some of the basic emotional needs are chronically unmet by parental figures. These patterns form during childhood and/or adolescence and manifest themselves in adulthood as attitudes, thoughts or emotions that are dysfunctional in relation to the situation experienced in the present.

We have summarized them in Table 1:

Empirical research on Schema Therapy has shown promising outcomes for BPD, with studies indicating significant improvements in personality pathology and quality of life. The experiential and relational components of Schema Therapy—particularly Chairwork and limited reparenting—demonstrate convergence with Gestalt’s emphasis on embodied contact and authentic therapeutic presence. Recent research suggests that emotion regulation strategies shared across DBT and Schema Therapy may represent common active ingredients [13], supporting the theoretical coherence of the integration proposed here.

### 4.2. Coping Strategies

There are three coping strategies that individuals can employ:

Surrender: behaving as if there were no alternative to the pattern;

Hypercompensation: behaving as if the opposite of the pattern were true;

Avoidance: avoiding both thinking about and experiencing situations that trigger the pattern.

### 4.3. Mode

A mode is a combination of various activated Early Maladaptive Schemas mixed with coping strategies; the concept of mode describes the emotional-cognitive-behavioural state in which the person finds themselves at a given moment. Functional modes promote positive adaptation, while dysfunctional modes are characterized by strategies that can culminate in states of distress, avoidance, or self-sabotaging behaviours.

In Gestalt work with patients with borderline personality disorder, the integration of Schema Therapy concepts significantly enriches the phenomenological understanding of the patient’s experience. Schema Therapy modalities find a natural correspondence with the Gestalt concept of figure/background, where different configurations of the Self emerge and recede in the phenomenological field depending on the contact activated.

**Figure 1:**
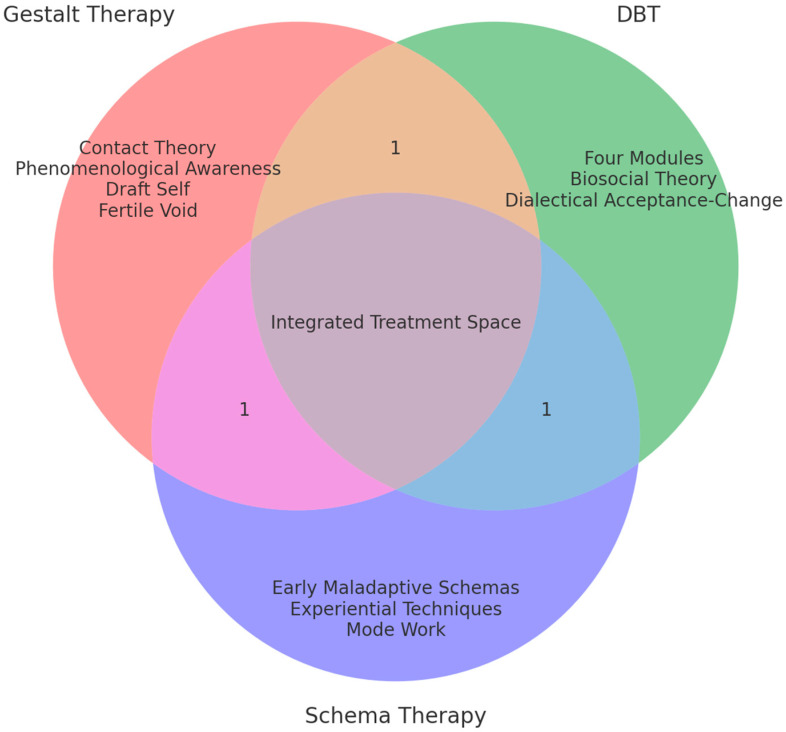
Conceptual Integration Model: Gestalt Therapy, DBT, and Schema Therapy for BPD.

Coping strategies—surrender, overcompensation, and avoidance—can be reinterpreted as creative ways for the organism to adapt, which, in the therapeutic here-and-now, manifest themselves through specific modes of contact or interruptions of contact itself. The Gestalt therapist, supporting moment-to-moment awareness, accompanies the patient in observing how these early patterns are actualized in the therapeutic relationship, not to analyze them cognitively but to experience them phenomenologically. This integration allows the anti-pathologizing approach of Gestalt to be maintained—recognizing patterns as creative adaptations of the patient in invalidating environments—while using the conceptualization of Schema Therapy to navigate the complexity of borderline configurations, promoting the emergence of more functional modes through the experience of authentic contact (Figure 1).

In the experience of the tolerable novelty of the therapeutic encounter, one can play with inventing new personal patterns, recognizing one’s own, modifying them, and tracing their boundaries [14].

E.g., To do after grounding

*Imagine entering a space that you recognize intimately. It is an environment that you feel is yours, even if it does not always give you peace of mind. This place is very familiar to you… it once offered you shelter, but now it seems to limit your movements. Perhaps it is the habit of always having to appear invulnerable… or the tendency to put the needs of others before your own. Observe this environment with curiosity. What colors characterize it? What atmosphere do you perceive? Are there any elements that attract your attention? Presences? Paintings or photos on the walls?*

*Now, in front of you, a passageway appears. This passageway invites you outside this space… toward an unexplored dimension. Move toward it calmly… Before crossing it, feel that you can decide… Do you want to stay in this familiar environment, or do you want to experience, even briefly, what it means to cross it?*

*You don’t need to have all the answers. It is enough to feel curiosity for something new. Cross the threshold.*

*Beyond it, you feel a refreshing breeze, brightness, spaciousness. Perhaps there is a little fear, but it is accompanied by a sense of openness. Keep in mind that you can always return to this dimension whenever you wish.*

*The choice is yours.*

## 5. Dialectical Interventions Between Acceptance and Change


*If a patient says to the therapist:*

*“The moon is made of cheese,” and the therapist replies:*

*“The moon and cheese are both yellow,”*

*we are witnessing a hermeneutic and clinical revolution.*
Giovanni Salonia

The paradoxical theory of Gestalt change and working with polarities naturally align with the dialectic (dià-legein meaning “to speak through,” but also “to gather” + tèchne, meaning “the art” of dialogue and bringing together) acceptance-change of DBT. Rather than denying the patient’s experience, therapists reconnect with “AND” statements that are perceptually verifiable and non-judgmental, keeping relational fields alive.

Examples of the application of this work include:

**Integrating chairs as drafts of oneself:** One chair expresses the punitive parental schema while the other embodies the vulnerable child.

**Dramaturgy:** Applied in Gestalt therapy by guiding patients to reimagine painful scenes and insert nurturing figures.

Research has shown how schema-focused imagery and DBT emotional regulation strategies effectively adapt to emotional regulation patterns, illustrating their compatibility with the embodied orientation of Gestalt [13].

## 6. Clinical Implementation

### 6.1. Session Structure and Process

The integrated approach maintains the non-protocol essence of Gestalt by incorporating structured elements:

#### 6.1.1. Pre-Contact

Establish awareness of the present moment and assess your current emotional state (look at the draft in the present moment with possible experiences of mindfulness, listening, storytelling, drawing, writing, etc.) to create a shared here and now.

#### 6.1.2. Start of Contact

Phenomenological tracking of emerging contact patterns, with particular attention to:

• Fluctuations in boundaries.
• Somatic indicators of dissociation or splitting.
• Interpersonal enactments within the therapeutic relationship.

#### 6.1.3. Full Contact

Recognize the domains [12] of the therapeutic relationship with a patient with BPD and support the integration process (Table 2).

#### 6.1.4. Post Contact

Support assimilation and discarding processes by emphasizing directionality (next).

### 6.2. Therapeutic Posture and Relationship

The therapist maintains a dual awareness:

Holding space for the Draft Self while containing phenomena at the process level.

To improve one’s therapeutic posture, the therapist’s embodied presence is the first essential element, referring to the clinician’s ability to maintain their emotional and physical grounding (countertransference) during the intense relational dynamics that characterize working with emotionally dysregulated patients [15]. This stable presence provides a safe container for the patient’s experience. The second component is phenomenological curiosity, which directs therapeutic attention toward the how of the patient’s symptomatic manifestations, rather than toward premature causal interpretations of the why. This shift in focus allows for a more immediate and concrete understanding of the patient’s lived experience. Finally, collaborative formulation is a mode of co-constructing meaning that integrates neurobiological understanding with subjective experience. When patients ask questions such as “Why am I so reactive?”, the therapist can respond with formulations that acknowledge both individual biological sensitivity and environmental influences on dysregulation, while simultaneously redirecting attention to the phenomenological analysis of the present moment: “You are biologically sensitive and your environment has not helped to regulate this—let’s explore how it manifests itself right here in our interaction.” This integration of presence, phenomenological curiosity, and collaboration allows the therapeutic process to be anchored in the immediacy of shared experience, facilitating co-constructed regulation processes.

### 6.3. Training Requirements and Supervision Framework

The implementation of this integrative model requires substantial clinical competence across three complex modalities, raising important questions about training pathways and quality assurance.

#### 6.3.1. Minimum Training Requirements

Therapists should possess: (1) foundational training in Gestalt therapy with supervised clinical experience in contact boundary work and phenomenological tracking; (2) certified training in DBT fundamentals, including the four skill modules and biosocial theory; (3) basic familiarity with Schema Therapy concepts, particularly early maladaptive schemas and experiential techniques.

#### 6.3.2. Supervision Architecture

We recommend a developmental supervision model that includes: (1) Phase 1 (6–12 months): Focus on maintaining Gestalt phenomenological stance while introducing DBT skills in a structured manner; (2) Phase 2 (12–24 months): Integration of schema-focused interventions with emphasis on avoiding cognitive over-interpretation; (3) Phase 3 (ongoing): Advanced work on managing epistemological tensions and developing flexible responsiveness.

Supervision should address not only technical integration but also the therapist’s capacity to hold multiple theoretical frameworks without rigidity. Group supervision formats that include peer review of session recordings can support this developmental process. Without adequate training infrastructure, there is risk of superficial eclecticism rather than coherent integration—a concern we take seriously in proposing this demanding model.

## 7. Clinical Illustration: Managing Emotional Dysregulation in Session

The following composite vignette illustrates the integrated approach during a moment of acute emotional dysregulation.

Context: Maria, a 28-year-old woman with BPD, arrives 15 min late, visibly agitated. She immediately accuses the therapist of “not caring” because he didn’t respond to her late-night text.

Phenomenological Tracking (Gestalt): The therapist notices Maria’s shallow, rapid breathing, clenched fists, and averted gaze—somatic indicators of splitting and potential dissociation.

Therapist intervention: “Maria, I notice your breathing is very quick right now, and you’re holding your hands tightly. Can you feel that?” [Grounding in embodied awareness]

Maria: “I don’t care about breathing! You don’t care about me!”

Dialectical Response (DBT-informed): “I hear that you’re feeling abandoned right now, and I want you to know I’m here with you now. Can we stay with what’s happening in your body for just a moment?” [Validation + redirection to present moment]

Maria: [Breathing slows slightly] “I just… I felt so alone last night.”

Schema-Focused Exploration: “That feeling of being alone… where do you notice it in your body right now?” [Maria places hand on chest] “Can you stay with that sensation? What does it need?”

Maria: [tears forming] “It needs… to know someone is there.”

Gestalt Experiment: “I’m right here, Maria. Can you see me? I’m not looking away. I’m here.” [Therapist maintains steady eye contact, embodying reliable presence] “What do you notice as you look at me?”

Maria: “You’re… you’re still here.” [Integration moment: experiencing relational continuity despite internal fragmentation]

Post-Contact Assimilation: “You came in feeling I didn’t care, and you just experienced me staying present with you even when you were angry. What is that like for you?”

This brief sequence demonstrates: (1) immediate phenomenological attention to somatic indicators; (2) dialectical validation without abandoning therapeutic structure; (3) schema activation made visible through embodied exploration; (4) Gestalt contact work that supports integration of the ‘Draft Self’ through lived relational experience.

## 8. Discussion

### Theoretical Consistency

The integration of the biosocial theory of DBT, mindfulness, and dialectical work between polarities significantly enriches the theoretical framework of Gestalt. Evidence from recent meta-analyses supports the efficacy of both DBT and Schema Therapy in reducing BPD symptomatology [16,17,18]. The biosocial lens of DBT, which places emotional dysregulation at the intersection of innate sensitivity and invalidating environments, finds phenomenological complementarity in Gestalt’s emphasis on moment-to-moment awareness of lived experience.

This integrative process [19] accompanies observation and reorganization that is tolerable for the patient and creates a minimalist relationship of contact with the therapist, maintaining an optimal distance for both that prevents retraumatization. We hypothesize that this integration may facilitate a conscious and dynamic outline of the “consequences of love” that can lead to regulating emotional processes without completely identifying with them, but observing them when they can modify the outline without destroying it.

From this perspective, the Self is no longer merely fragmented but becomes a form of functioning in the environment and, consequently, in the relationship between patient and therapist, in line with the Gestalt principle of Self theory, according to which the Self is not a static entity but a dynamic process that emerges from the creative contact between organism and environment and is articulated through three interconnected functions that operate at different times: Es function—Represents the receptive dimension of experience, characterized by a passive quality that concerns “what happens to us” beyond our conscious will. Ego Function—Allows us to modulate the degree of openness or closure in contact, deciding whether to accept, reject, or limit what emerges from experience. Personality Function—Constitutes the internal representation of the self, deriving from the integration of experiences lived throughout existence. It is the identity substrate that allows us to recognize ourselves over time and to give continuity and meaning to our experiences.

We propose that borderline presentations may be particularly well-suited to Gestalt therapy’s anti-neurotic epistemology, as symptoms can be understood as creative adaptations rather than pathological deficits—though this theoretical affinity requires empirical examination. Rather than pathologizing symptoms, they are recognized as creative adaptations of the organism to difficult situations. Integration with DBT tools does not aim to eliminate “negative” emotions, but to develop a more fluid and conscious relationship with the entire emotional spectrum.

As Greenberg points out, “changing emotions with emotions” [20], the therapeutic process is not based on suppression or control, but on transformation through access to more adaptive and authentic emotional resources. This principle resonates deeply with the Gestalt approach of supporting organismic spontaneity and the intrinsic wisdom of the self-regulation process [21].

## 9. Conclusions

This integrated approach is not limited to simply modulating emotional reactions, but aspires to a deeper and ontologically significant transformation: the metamorphosis of the unbridgeable void that characterizes the borderline experience into what Gestalt defines as a “Fertile Void” [22].

While the pathological void of BPD is experienced as devastating absence, identity fragmentation, and existential horror vacui, the Fertile Void represents a potential space for creativity, a field open to emerging possibilities [23], and a generative terrain for self-realization [24]. Through the integration of biosocial validation, restructuring of maladaptive schemas, and embodied presence in the here-and-now, the patient can gradually experience this emptiness no longer as an abyss to be compulsively filled, but as a space for conscious breathing, a creative pause between stimulus and response, a fertile silence from which new gestalts of meaning can emerge.

This transmutation of emptiness—from an experience of annihilation to a therapeutic resource—is perhaps the most distinctive contribution of this integrated model, offering patients with BPD not only symptomatic stabilization but access to an existential dimension of fullness paradoxically rooted in the conscious acceptance of their own inner space [25].

### 9.1. Practical Implications for Clinical Training and Practice

Beyond its theoretical contributions, this integrative framework offers several concrete implications for clinical training and supervision. Training programmes seeking to implement this model should prioritize three developmental competencies: (1) *Phenomenological precision*—the capacity to track moment-to-moment somatic and relational indicators without premature interpretation; (2) *Dialectical flexibility*—the ability to hold validating and change-oriented stances simultaneously without collapsing into either pole; and (3) *Epistemological humility*—recognizing when to employ structured interventions versus when to trust organismic process.

Supervision architectures should incorporate video-modelling, live session observation or recording review to identify moments where therapists default to cognitive interpretation rather than embodied exploration, or conversely, where phenomenological tracking could benefit from structured DBT skill application. Peer consultation groups using the five therapeutic domains (Table 2) as reflective frameworks can support clinicians in recognizing their characteristic response patterns with borderline presentations—whether tending toward over-containment or under-structure.

For practicing clinicians trained primarily in one modality, we recommend gradual integration rather than wholesale adoption: DBT-trained therapists might begin by incorporating 2–3 min grounding experiments before skills coaching; Gestalt practitioners might systematically introduce distress tolerance psychoeducation while maintaining phenomenological stance; Schema therapists could emphasize embodied awareness during Chairwork rather than exclusively cognitive processing. The integration succeeds not through theoretical mastery but through lived experimentation within one’s existing clinical competence, allowing the model to emerge organically from practice rather than being imposed as protocol.

### 9.2. Limitations

This framework carries important limitations. First, as a theoretical synthesis without empirical testing, claims about therapeutic efficacy remain speculative. Second, the integration assumes high therapist competence across multiple modalities, which may limit real-world applicability. Third, we have not addressed how this model adapts across diverse cultural contexts or comorbid presentations (e.g., BPD with substance use disorders or psychotic features).

Fourth, the epistemological tensions between approaches—particularly Gestalt’s anti-pathologizing stance and the medical-model origins of DBT/Schema Therapy—are managed pragmatically here but may prove more intractable in practice. Finally, the heavy reliance on Gestalt-oriented literature may limit the model’s accessibility to clinicians trained primarily in cognitive-behavioural traditions. These limitations underscore the preliminary nature of this proposal.

### 9.3. Future Direction

This theoretical framework opens several promising avenues for development. First, empirical validation through systematic case studies and eventually randomized controlled trials comparing this integrated approach with standard DBT or Schema Therapy would provide crucial evidence for its clinical efficacy. Process research examining specific mechanisms—such as how embodied Gestalt experiments facilitate schema change, or how phenomenological tracking enhances emotional regulation—could illuminate the active ingredients of integration.

Second, the development of structured training programmes and treatment fidelity measures would support wider dissemination while maintaining theoretical coherence. Third, exploration of how this model adapts across cultural contexts and diverse clinical presentations beyond classic BPD would test its flexibility and generalizability.

Finally, theoretical dialogue with emerging frameworks such as the Power Threat Meaning Framework and neuroscience-informed psychotherapy could further enrich this integrative paradigm. We view this paper as an invitation to such collaborative inquiry rather than a definitive statement.

### 9.4. Methodological Considerations for Empirical Validation

Several research designs could systematically test this integrative framework.

**Single-case experimental designs (SCEDs)** using multiple baseline or alternating treatment formats would allow fine-grained examination of how specific integration elements (e.g., embodied grounding before DBT skills training) impact measurable outcomes such as dissociative symptoms or emotional dysregulation episodes. These designs preserve the idiographic, process-oriented nature of Gestalt work while providing experimental rigour.

**Qualitative phenomenological studies** employing Interpretative Phenomenological Analysis (IPA) or grounded theory could explore how patients with BPD experience the integration—particularly whether the dialectical stance and embodied focus address the existential dimensions (identity coherence, Fertile Void transformation) that quantitative symptom measures may overlook. Interviews focusing on patients’ lived experience of the “Draft Self” concept and its therapeutic utility would illuminate whether this theoretical construct resonates with subjective phenomenology.

**Dismantling studies** comparing standard DBT, standard Gestalt therapy, and the integrated model across matched samples could identify whether the integration produces additive or synergistic effects. Process measures tracking therapeutic alliance rupture-repair sequences, use of contact boundary language, and frequency of embodied experiments versus cognitive interventions would illuminate mechanisms of change.

Finally, **task analysis** methodologies—microanalytic examination of session transcripts identifying “change events”—could map the exact sequencing of interventions (e.g., phenomenological tracking → validation → schema identification → embodied experiment) that facilitate integration moments. This approach aligns with both Gestalt’s process-orientation and contemporary psychotherapy research emphases on mechanisms rather than manualized protocols.

## Figures and Tables

**Table 1:** Early maladaptive schemas and domains. This table represents an original synthesis developed by the authors, integrating concepts from Spagnuolo Lobb’s ‘now-for-next’ framework [12] with clinical observations from borderline treatment contexts. The five domains are proposed as heuristic guidelines rather than empirically validated stages.

Domain	Scheme	Description	Gestalt Integration
1. Detachment and rejection	Emotional deprivation	The belief that fundamental emotional needs cannot be met by others.	Contact boundary work: Explore how deprivation schema manifests as retroflection (turning needs inward, self-denial) or deflection (minimizing or dismissing needs). Gestalt experiments focus on articulating and voicing needs directly in the present therapeutic relationship, allowing the patient to experience the possibility of contact rather than perpetual deprivation. The therapist’s embodied presence offers a corrective relational experience.
	Abandonment/Instability	The expectation that relationships with others are unstable and may end.	Draft Self preservation: Abandonment fears reflect the fragility of the provisional “Draft Self” that cannot sustain relational disruption without existential collapse. The therapist’s consistent, non-reactive presence—surviving the patient’s relational storms without withdrawal—offers lived evidence of relational continuity. Experiments explore the phenomenology of “staying” versus “leaving” through body awareness and boundary work.
	Distrust/Abuse	The expectation that others will hurt, humiliate or deceive us.	Projection and introjection work: Distrust often involves projecting past abusive experiences onto current relationships. Gestalt work focuses on differentiating “then” from “now” through phenomenological inquiry: “What are you experiencing right herewith me?” The therapist models transparency and authenticity, making the therapeutic process visible and co-constructed, thereby reducing projective distortions.
	Social isolation	A feeling of not belonging to any community.	Field theory perspective: Social isolation is reframed as disrupted organism-environment contact. Rather than an internal deficit, it represents a creative adjustment to environments that failed to provide belonging. Gestalt group work and experiments exploring “reaching out” gestures can help patients experience moments of authentic connection and membership in the therapeutic community.
	Inadequacy/Shame	The belief that one cannot be loved because one is imperfect, inferior, or bad.	Shame as interrupted contact: Shame represents a profound interruption of contact with self and other. Gestalt work involves tracking the embodied experience of shame (body posture, gaze aversion, voice quality) and supporting gradual exposure to being “seen” by the therapist without withdrawal. The paradoxical theory of change suggests that accepting shame (rather than fighting it) creates space for transformation.
2. Reduced autonomy	Bankruptcy/Failure	The belief that one does not have sufficient skills to achieve results similar to others.	Organismic self-regulation: Reframe “failure” as interrupted contact with innate competence and wisdom. Gestalt experiments support small, achievable autonomous choices in session, validating emerging agency and resourcefulness. The focus shifts from comparison with others to reconnection with one’s own organismic capacity for creative adjustment and problem-solving.
	Dependency/Incompetence	A feeling of being powerless and unable to function independently.	Support function development: Dependency schemas involve confluence (over-merging with others) and loss of the ego function (capacity to make choices). Experiments focus on micro-moments of independent decision-making: “What do you want right now?” The therapist supports differentiation while remaining available, embodying the dialectic of autonomy and connection.
	Vulnerability to damage	The expectation that the world is full of dangers and that we do not have the resources to deal with them.	Grounding and embodiment: Vulnerability to harm involves chronic activation of survival responses and disconnection from present safety. Gestalt grounding techniques (feet on floor, breath awareness, sensory orientation) help patients experience present-moment safety. Experiments explore “risk assessment in real time”—differentiating actual from imagined threat through phenomenological investigation.
	Entanglement/Enmeshment	Excessive emotional involvement in the lives of one or more loved ones, fusion of identity.	Contact boundary clarification: Entanglement represents confluence—the loss of boundary between self and other. Gestalt boundary work focuses on the phenomenological question: “Where do I end and you begin?” Experiments using spatial distance, voice differentiation, and body awareness help patients experience their own separateness while maintaining connection, resolving the false dichotomy of fusion versus isolation.
3. Lack of rules	Entitlement/Grandiosity	Believing in one’s own superiority, having special privileges or being above the rules.	Deflection and narcissistic structure: Entitlement can represent deflection from underlying shame or fragility. Gestalt work involves gentle confrontation through phenomenological feedback: “I notice when I set a limit, you respond as if rules don’t apply to you. What happens in your body right now as I say this?” The focus is on exploring the protective function of grandiosity rather than moralizing.
	Insufficient self-control/Self discipline	Recurring difficulties with self-control, emotional management, and frustration tolerance.	Integration with DBT skills: Lack of self-regulation is understood as impaired contact with the Es function (impulses) and Ego function (choice-making). Gestalt experiments incorporate DBT distress tolerance skills—taught not as behavioural control but as expanding the “space between impulse and action.” The therapist supports awareness of the exact phenomenological moment when choice becomes possible.
4. Excessive attention to the needs of others	Subjugation	Giving up one’s desires, believing that the will of others takes priority in order to avoid negative consequences.	Retroflection and aggression work: Subjugation involves retroflecting healthy aggression (self-assertion) and maintaining confluence to avoid conflict. Gestalt experiments invite the patient to speak their truth in session, even if it conflicts with the therapist’s suggestions. The therapist’s non-defensive response demonstrates that healthy assertion does not destroy relationships—challenging the core schema assumption.
	Self-sacrifice	The belief that one must constantly satisfy the needs of others at the expense of one’s own.	Reclaiming organismic needs: Self-sacrifice represents chronic deflection from one’s own needs and over-identification with caretaking roles. Gestalt work focuses on the phenomenological question: “What do you need right now?” Experiments may involve role reversals (e.g., chair work where the patient receives care rather than gives it) to experientially challenge the schema.
	Approval-seeking/Recognition-seeking	Basing self-esteem on social acceptance and approval, on which personal value depends.	Validation from within: Approval-seeking reflects interrupted contact with intrinsic self-validation. Gestalt experiments explore the embodied experience of self-approval: “Can you place your hand on your heart and say ‘I see you’?” The therapist models authentic appreciation (not praise) and supports the patient in tracking internal rather than external validation cues.
5. Hypercontrol and emotional inhibition	Emotional inhibition	A reduction in emotional expression and genuine feelings in order to avoid rejection	Expression experiments: Emotional inhibition involves chronic retroflection (holding back expression) and desensitization. Gestalt work uses experiments in graduated emotional expression—perhaps starting with naming emotions, then using voice tone variation, then full embodied expression. The therapist’s capacity to receive and “survive” emotional expression without rejection is therapeutic.
	Unrelenting standards/Hypercriticalness	The belief that extremely high standards must be met in order to gain approval.	Perfectionism as top-dog/underdog split: Perfectionism reflects an internal split between the critical “top dog” (demanding perfection) and the overwhelmed “underdog” (never good enough). Gestalt chair work externalizes this split, allowing dialogue between parts. The therapeutic stance emphasizes “good enough” contact rather than perfect performance, embodying the paradoxical theory of change.
	Negativity/Pessimism	A view of life focused on the negative aspects, on what can go wrong.	Figure/ground reversal: Pessimism involves selective attention to negative figures while positive experiences recede into background. Gestalt experiments deliberately shift attention: “What is going well in this moment?” or “Notice three things in this room that bring you pleasure.” This is not positive thinking but phenomenological rebalancing of the perceptual field.
	Punitiveness	The belief that people should be severely punished for their mistakes.	Introjection work: Punitiveness often represents an introjected parental or cultural voice. Gestalt work involves identifying and “chewing” (critically examining) these harsh introjects: “Whose voice is this?” Experiments may involve speaking the punitive voice aloud, then responding from an alternative, compassionate perspective, creating internal dialogue rather than monologue.

**Table 2:** **Domains of the therapeutic relationship with a patient with BPD.** This table represents an original synthesis developed by the authors, integrating concepts from Spagnuolo Lobb’s *now-for-next* framework [12] with clinical observations from borderline treatment contexts.

*Domains*	*Domain 1*	*Domain 2*	*Domain 3*	*Domain 4*	*Domain 5*
***Name***	A confident, clear, and non-manipulative ethical stance.	Capture the now-for-next in the patient’s relational difficulties.	Explain the elements of shared reality.	Support self-regulation in the face of primitive defences.	Containing borderline suffering through countertransference.
***Therapist skills***	Containment capacity; Ethical clarity; No manipulation.	Capture the tension of being fully present with the other person, despite aggressive and demeaning language.	Create a bridge between the current reaction and painful relationship patterns.	Developing a therapeutic language that captures the desire for integration between affection for others and autonomy.	Listening to countertransference emotions and their therapeutic contextualization.
***Therapeutic objectives***	Support the patient’s primary intention to rely on that therapist.	The patient experiences the ability to preserve the outline of themselves with the other, despite the ambivalence that causes him to lose his sense of integrity.	Experience the coherence between past pain and current reaction. Feel the therapist’s closeness in the attempt to integrate conflicting parts.	Experiencing both the ability to reach out to others and perceptual autonomy.	Validate the patient’s desperate experience and cope with the split with less anxiety and reactivity.

## Data Availability

No new data were created or analyzed in this study. Data sharing is not applicable to this article.
